# Effects of Different Forms of Selenium in Human Umbilical Cord Mesenchymal Stem Cells

**DOI:** 10.3390/biomedicines13122861

**Published:** 2025-11-24

**Authors:** Beibei Ni, Cuiping Li, Huizhu Lin, Wenjie Chen, Ruixuan Xu, Huali Li, Xiaoyan Chen, Jianxi Lu, Fan Yang

**Affiliations:** 1 Vaccine Research Institute, Cell-Gene Therapy Translational Medicine Research Centre, The Third Affiliated Hospital of Sun Yat-sen University, Guangzhou 510630, China; nibb@mail.sysu.edu.cn; 2 Biotherapy Centre, The Third Affiliated Hospital of Sun Yat-sen University, Guangzhou 510630, Chinachenwj5@mail.sysu.edu.cn (W.C.);; 3 Xinjiang Stem Cells Special Plateau Disease Engineering Technology Research Center, The First People’s Hospital of Kashi, The Affiliated Kashi Hospital of Sun Yat-sen University, Kashi 844000, China; 4 Department of Infectious Diseases, The Third Affiliated Hospital of Sun Yat-sen University, Guangzhou 510630, China

**Keywords:** mesenchymal stem cells, CS-SeNPs, Na_2_SeO_4_, cell proliferation, cell senescence

## Abstract

**Background:** Mesenchymal stem cells (MSCs) have shown positive therapeutic effects on various diseases; however, their functionality can decline during in vitro expansion. Selenium (Se) supplementation has emerged as a strategy for enhancing MSC culture. This study evaluated the effects of different forms of selenium (Na_2_SeO_4_, SeMet, ebselen, and chitosan-coated selenium nanoparticles (CS-SeNPs)) on the biological functions of MSCs. **Methods:** Human umbilical cord-derived MSCs (HUC-MSCs) were cultured in media supplemented with various selenium compounds at specific concentrations. To investigate their biological effects, we assessed cell proliferation, morphology, surface marker expression, and differentiation potential. Furthermore, to elucidate the underlying mechanisms, we analyzed key markers of cellular senescence, including *p16*, *p21*, *IL-6*, *IL-8*, *p27*, *p53*, and reactive oxygen species (ROS) levels. **Results:** All the selenium treatments promoted hUC-MSC proliferation at specific concentrations. CS-SeNPs and Na_2_SeO_4_ exhibited relatively high bioavailability, whereas ebselen and SeMet demonstrated relatively low toxicity. The optimal concentration (0.5 μM CS-SeNPs or 0.25 μM Na_2_SeO_4_) significantly enhanced proliferation without altering the hUC-MSC morphology, phenotype, or differentiation capacity. Both CS-SeNPs and Na_2_SeO_4_ effectively promoted hUC-MSC proliferation and reduced the senescence of hUC-MSCs by downregulating key senescence-related effectors: the cell cycle inhibitors *p16*, *p21*, *p27*, and *p53*; and the levels of ROS and senescence-associated secretory phenotype factors (*IL-6* and *IL-8*). **Conclusions:** Selenium supplementation is an effective strategy for improving MSC expansion and alleviating senescence. The beneficial effects are dependent on the specific selenium compound used, with CS-SeNPs and Na_2_SeO_4_ showing particularly strong potential for enhancing the bioavailability and function of hUC-MSCs during in vitro cultivation.

## 1. Introduction

In recent decades, stem cell therapy has attracted increasing interest from researchers because of its potential for treating many diseases that are refractory to multiple advanced treatments [1,2]. Mesenchymal stem cells (MSCs), which are derived from the stroma of various tissues, such as the bone marrow, umbilical cord and adipose tissue, hold great promise for the development of novel therapeutics [3,4]. Numerous studies have shown that the transplantation of MSCs has beneficial therapeutic effects in a range of diseases [5,6]. To date, thousands of clinical trials involving human MSCs have evaluated their potential for clinical application and effectiveness worldwide [7]. A phase III randomized clinical trial demonstrated that MSC infusion after acute myocardial infarction significantly reduces heart failure risk and improves cardiac function [8]. Recent research by Shi et al. [9] in cirrhosis highlights that determining the optimal dosage and administration protocol for MSCs remains a central challenge for clinical translation. Preserving the “youthful” phenotype and function of MSCs is thus critical for producing the high-quality cells required to realize their full clinical potential.

Cell-based therapies require a substantial number of cells, which typically need to be extensively expanded in vitro. However, prolonged culture of MSCs leads to cellular phenotypic aging, a critical issue that severely constrains the production of high-quality cell products. Senescent MSCs exhibit not only reduced proliferative capacity but also diminished multilineage differentiation potential, compromised immunomodulatory function, and adoption of a proinflammatory senescence-associated secretory phenotype, collectively undermining their therapeutic efficacy. These functional declines highlight the urgent need for strategies to delay senescence and preserve the functional potency of MSCs. Researchers have used a variety of strategies, such as the optimal source of stem cells and the improved MSC culture medium, to enhance the impaired functionality of stem cells, which has improved certain aspects of cell therapy [10,11]. For instance, Tee et al. [12] demonstrated that L-ascorbic acid-2-phosphate supplementation significantly reduces cellular senescence and improves the chondrogenic potential of MSCs, resulting in increased critical metabolic and functional properties. These findings underscore the value of chemical preconditioning in preserving MSC quality during manufacturing.

Selenium (Se), an essential microelement for humans, plays a crucial role in various physiological functions. A moderate intake of selenium can significantly improve immune function and positively influence human health. However, poor selenium levels may lead to several diseases such as Keshan disease [13,14]. Selenium, primarily through its incorporation into selenoproteins such as glutathione peroxidases and thioredoxin reductases. These enzymes combat cellular senescence by neutralizing reactive oxygen species and modulating key signaling pathways to mitigate oxidative stress and its associated damage [15,16]. However, the specific application of selenium as a culture supplement to combat MSC senescence remains underexplored, and its effects on critical MSC properties during long-term culture are not well defined. Standard MSC culture media provide essential nutrients but lack specific components to combat replication-induced senescence and functional decline. This study aims to systematically evaluate whether selenium supplementation in standard culture medium can effectively delay MSC senescence, preserve their functional potency, and ultimately enhance their therapeutic quality.

Selenium is generally divided into inorganic selenium (e.g., Na_2_SeO_4_) and organic selenium. Organic selenium includes both naturally occurring forms, such as selenomethionine (SeMet) and selenocysteine, as well as synthetic molecules, such as ebselen—a glutathione peroxidase mimetic with demonstrated clinical efficacy in inflammatory arthritis [17]. These different chemical forms exhibit substantial variations in their biological properties. Inorganic selenate (Na_2_SeO_4_), while naturally abundant, has greater toxicity and lower bioavailability than organic forms. While organic selenium compounds show improved absorption and incorporation into selenoproteins, they may accumulate nonspecifically in tissues. Most notably, selenium nanoparticles (SeNPs) are highly efficient molecular compounds with greater antioxidant activity and lower toxicity than regular selenium. Numerous compounds, such as polysaccharides, amino acids, and peptides, have been used to modify the surface of SeNPs [18,19]. Chitosan (CS) is an alkaline polysaccharide that is characterized by its good biocompatibility, non-toxic nature, low immunogenicity, and biodegradability [20]. Compared with both inorganic and simple organic selenium compounds, SeNPs modified with CS (CS-SeNPs) can exhibit superior properties, including enhanced stability, reduced toxicity, and improved cellular uptake [21]. Furthermore, its excellent biocompatibility and biodegradability minimize its cytotoxic effects, while its matrix structure modulates selenium release, protecting cells from acute oxidative stress associated with bare SeNPs [22,23]. These characteristics make CS-SeNPs particularly suitable for maintaining MSC functionality during long-term culture, as they ensure consistent colloidal stability, increase selenium bioavailability, and may provide synergistic antioxidant effects through the bioactivity of chitosan. These fundamental differences in pharmacokinetics and biosafety profiles guided our selection of diverse selenium forms for comparative assessment in MSC culture.

Human umbilical cord-derived MSCs (hUC-MSCs) were selected for this study because of their compelling advantages for translational applications, including abundant availability, robust proliferative capacity in vitro, and low immunogenicity suitable for allogeneic transplantation. These properties make hUC-MSCs an ideal cell source for future therapeutic development, which is why we used hUC-MSCs in our subsequent experiments.

The biological efficacy of selenium in hUC-MSCs is associated with its chemical form, and it has not yet been determined which form of selenium has better effects. On the basis of their unique properties, we hypothesized that CS-SeNPs would more effectively enhance hUC-MSC proliferation, reduce oxidative stress, and ameliorate senescence-associated phenotypes compared to their inorganic and organic counterparts. The present study was therefore designed to systematically evaluate the impact of different selenium forms in the process of hUC-MSC cultivation, aiming to establish an optimized protocol for producing a reliable and robust cell source for clinical applications.

## 2. Materials and Methods

### 2.1. Isolation and Culture of hUC-MSCs

The use of the hUC-MSCs used in this study was approved by the Clinical Research Ethics Committee of the Third Affiliated Hospital of Sun Yat-sen University (Approval number: SYSUTHEC RG2024-148-01). All umbilical cord samples (n = 5) were collected from healthy mothers with informed consent. For hUC-MSC isolation, blood vessels and the umbilical cord epithelium were removed from the umbilical cord. The obtained Wharton’s jelly was subsequently cut into tissue blocks of 2–3 mm^3^ and attached to culture dishes. The cells were collected and cultured with hUC-MSC serum-free medium (YOCON, Beijing, China) in a 5% CO_2_, 37 °C incubator. When the cells reached 80% to 90% confluence, the hUC-MSCs were passaged at a dilution ratio of 1:3 with 1× stem cell gentle digestion enzyme (YOCON, China) for 2–5 min following the manufacturer’s instructions. All the hUC-MSC cultures were routinely tested for mycoplasma contamination and sterility. Mycoplasma testing was performed via a PCR-based method with primers (forward: 5′-TGCACCATCTGTCATTCTGTTAAC-3′; reverse: 5′-GGAGCAAACAGGATTAGATACCCT-3′) and SYBR Safe DNA gel stain (Invitrogen, Carlsbad, CA, USA). Sterility was confirmed by inoculating culture supernatants into the zLABSTAR EX (Brea, CA, USA) automated blood culture system and monitoring microbial growth for 5 days. All cultures used in the experiments were confirmed to be negative for both mycoplasma and microbial contamination.

### 2.2. Cell Culture and Selenium Compound Treatment

HUC-MSCs at passage 3 were suspended in MSC medium with or without combinations of the following selenium compounds: CS-SeNPs, ebselen, Na_2_SeO_4_ and SeMet. Ebselen, Na_2_SeO_4_ and SeMet were purchased from Sigma-Aldrich (St. Louis, MO, USA) and dissolved in sterile PBS buffer. CS-SeNPs were synthesized as described by Liu et al. [24]. The culture medium was changed every two days. Cells from at least three different hUC-MSC donors were used for all the experiments. CS-SeNPs (0.5 μM) and Na_2_SeO_4_ (0.25 μM) were selected as the optimal concentrations for further experiments. The morphology of the cells across all test groups was examined via optical microscopy (Carl Zeiss MicroImaging GmbH, Oberkochen, Germany). The evaluated parameters included cell size and granularity and the proportion of cells with a classical spindle-shaped, fibroblast-like morphology versus a flattened, enlarged, or irregular shape.

### 2.3. Cell Counting Kit-8 Assays

The cells were plated in 96-well plates at a density of 1000 cells per well and then treated with CCK-8 solution (Dojindo Laboratories, Kumamoto, Japan). The absorbance at a wavelength of 450 nm was subsequently measured via a multifunctional microplate reader (Tecan200, Männedorf, Switzerland) to assess cell viability. Each experiment was performed with three independent biological replicates.

### 2.4. Flow Cytometry

To examine the expression of hUC-MSC phenotypic markers, 2 × 10^6^ cells were stained with specific antibodies and incubated in the dark for 30 min at room temperature following the manufacturer’s instructions. Flow cytometry was performed with the following antibodies: anti-CD105 (A07768), anti-CD90 (A07768), anti-CD73 (344003), anti-CD45 (A07768), anti-CD34 (A07768), anti-CD19 (A07768), anti-CD11B (IM0530), and anti-HLA-DR (A07768) were obtained from Beckman. Flow cytometry was performed with a flow cytometer from Beckman Coulter, Inc. (Brea, CA, USA). The experiment was performed with three independent biological replicates.

### 2.5. EdU Assays

Cell proliferation was measured via an EdU assay kit (RiboBio, Guangzhou, China), according to the manufacturer’s protocol. Briefly, cells were plated at 4 × 10^3^ cells per well in 96-well plates and cultured for 48 h. The cells were subsequently treated with 50 mM EdU for 2 h at 37 °C. Then, the cells were washed with PBS and fixed with 4% formaldehyde at room temperature for 30 min. The DNA contents of the cells were stained with 100 µL of Hoechst 33342 (RiboBio, Guangzhou, China) for 30 min and observed under a fluorescence microscope. The experiment was performed with three independent biological replicates.

### 2.6. ROS Analysis

The intracellular ROS levels were measured using the fluorescent probe 2′,7′-dichlorodihydrofluorescein diacetate (DCFH-DA) with an ROS assay kit (Beyotime, Shanghai, China) according to the manufacturer’s instructions. HUC-MSCs were resuspended at a density of 1 × 10^6^ cells/mL and treated with 10 μM DCFH-DA in a 37 °C cell culture incubator for 20 min. The cells were washed three times with serum-free cell culture medium to thoroughly remove DCFH-DA. The fluorescence of the cells was then measured via flow cytometry (Beckman Coulter, Brea, CA, USA) at excitation and emission wavelengths of 488 nm and 525 nm, respectively. The experiment was performed with five independent biological replicates.

### 2.7. Quantitative Real-Time PCR Assay (qPCR)

TRIzol Reagent was used for the extraction of RNA from the cells in our study (Thermo Fisher Scientific Inc., Waltham, MA, USA). Subsequently, reverse transcription was executed by employing the Evo M-MLV RT Kit with gDNA Clean (Accurate Biology, Changsha, China). ChamQ SYBR qPCR Master Mix (Vazyme, Nanjing, China) was utilized for the qPCR analysis. β-actin was utilized as the internal control gene for normalization in each qPCR assay. The relative expression of genes was determined with the comparative cycle threshold (CT) method, specifically the 2^−ΔΔCT^ method [25]. All qPCR analyses were performed with at least three independent biological replicates. The forward and reverse PCR primers used are listed in Table 1.

### 2.8. MSC Adipogenic Differentiation and Osteogenic Differentiation Analysis

For adipogenic differentiation, hUC-MSCs were seeded at a density of 2 × 10^4^ cells per well in six-well plates. When the cells reached 90% to 100% confluence, the hUC-MSCs were stimulated with an MSC adipogenic differentiation kit (Cyagen Biosciences, Guangzhou, China) according to the manufacturer’s instructions. The adipogenic differentiation medium was changed every 2 to 4 days. After 14 days of adipogenic induction, the cells were washed with PBS and fixed with 4% paraformaldehyde for 1 h. The induced cells were subsequently stained with Oil Red O. Stained cells were imaged using a Leica microscope (Leica, Wetzlar, Germany) and the staining intensity was quantified with ImageJ (version 1.54g, Bethesda, MD, USA) software.

For osteogenic differentiation, hUC-MSCs were seeded into six-well plates and cultured for 12 h at a density of 2 × 10^4^ cells per well. When the cells reached 70% confluence, the medium was replaced with hUC-MSC osteogenic differentiation medium (Cyagen Biosciences, Guangzhou, China) for 21 days, and the medium was refreshed every 3 days. Subsequently, the cells were washed with PBS and fixed with 4% paraformaldehyde for 1 h. Then, Alizarin Red S staining solution was added, and the cells were observed under a microscope. The staining intensity of the cells was assessed by quantification using ImageJ software. Each experiment was performed with three independent biological replicates.

### 2.9. Senescence-Associated β-Galactosidase (SA-β-Gal) Staining Assay

The SA-β-gal staining assay was carried out using an SA-β-gal Staining Kit from Beyotime according to the manufacturer’s protocol. Briefly, cells were plated at 1 × 10^5^ cells per well in 6-well plates and cultured for 48 h. The cells were subsequently fixed with a 4% paraformaldehyde solution for 15 min. These cells were subsequently incubated with SA-β-gal staining solution overnight in a 37 °C cell culture incubator and observed under an inverted microscope (Leica, Wetzlar, Germany). The cells exhibiting a dark blue color were identified as positive for the staining. The experiment was performed with three independent biological replicates.

### 2.10. Statistical Analysis

Experimental data analysis was conducted using Prism version 5.0 (GraphPad Software, La Jolla, CA, USA), employing Student’s *t*-test and one- or two-way ANOVA. All data were assessed for normality (Shapiro–Wilk test) and homogeneity of variances (Levene’s test) prior to parametric analysis. For the ANOVA models, Tukey’s honestly significant difference (HSD) post hoc test was applied for multiple comparisons between groups. Continuous data are presented as the means ± standard deviations (SDs) from at least three independent biological replicates. A significance level of 0.05 was chosen to determine statistical significance.

## 3. Results

### 3.1. Choice of Selenium Compounds for Improving the Culture of hUC-MSCs

**Figure 1:**
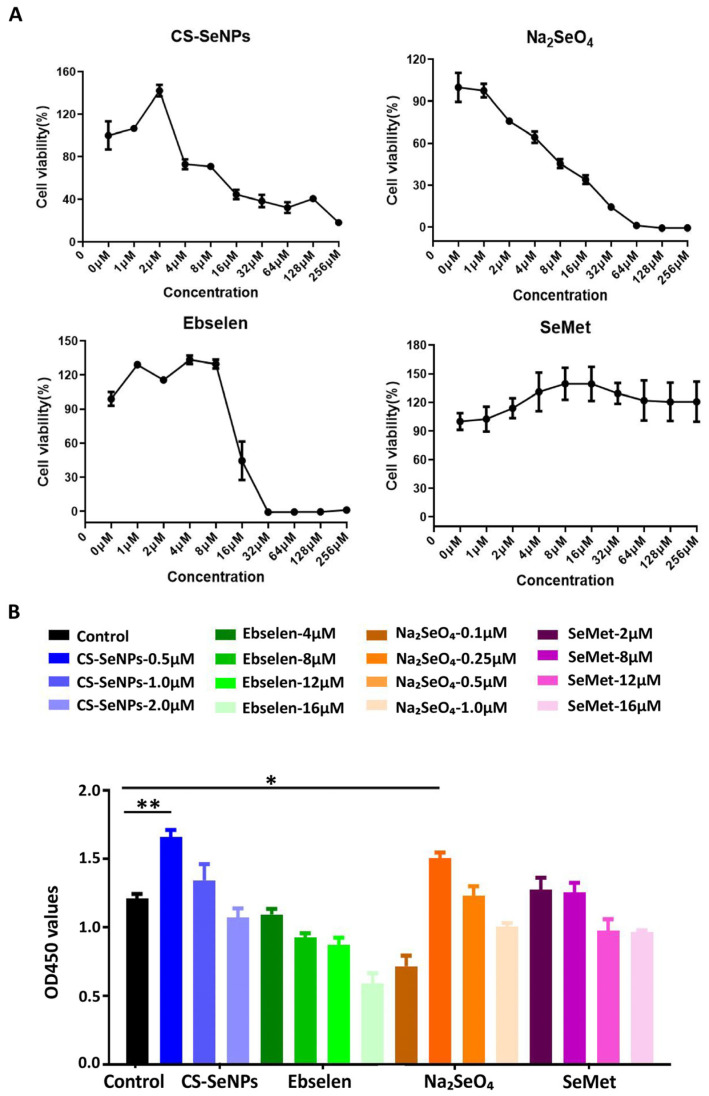
Choice of selenium compounds for improving the culture of hUC-MSCs. (**A**) CCK8 assays were performed to compare the proliferation rates of hUC-MSCs cultured with different concentrations of selenium. (**B**) CCK8 assays were used to detect the proliferation rates of the hUC-MSCs. * *p* < 0.05, ** *p* < 0.01. The data are from three independent biological replicates (n = 3) and are presented as the means ± SDs.

To investigate the optimal concentrations of different selenium compounds, CCK8 assays were conducted to compare the proliferation rates of hUC-MSCs cultured with different concentrations of the different selenium compounds (0 μM, 1 μM, 2 μM, 4 μM, 8 μM, 16 μM, 32 μM, 64 μM, 128 μM and 256 μM) [26,27,28]. As shown in Figure 1A, CS-SeNPs, ebselen and SeMet promoted cell proliferation to a certain degree at concentrations above 1 μM. However, 2 μM or higher concentrations of CS-SeNPs, 1 μM or higher concentrations of Na_2_SeO_4_, 8 μM or higher concentrations of ebselen and 32 μM or higher concentrations of SeMet have been shown to be toxic to hUC-MSCs and cause cell death. We further optimized the concentrations of these selenium compounds, with CS-SeNPs (0 μM, 0.5 μM, 1.0 μM and 2 μM), Na_2_SeO_4_ (0 μM, 0.1 μM, 0.25 μM, 0.5 μM and 1.0 μM), ebselen (0 μM, 4 μM, 8 μM, 12 μM and 16 μM) and SeMet (0 μM, 2 μM, 8 μM, 12 μM and 16 μM). Compared with those in the control group, the proliferation rates of hUC-MSCs were significantly increased following treatment with 0.5 μM CS-SeNPs or 0.25 μM Na_2_SeO_4_ after incubation for 72 h (Figure 1B). Thus, 0.5 μM CS-SeNPs and 0.25 μM Na_2_SeO_4_ were selected as the optimal concentrations for further experiments.

### 3.2. Basic Characterization of hUC-MSCs

**Figure 2:**
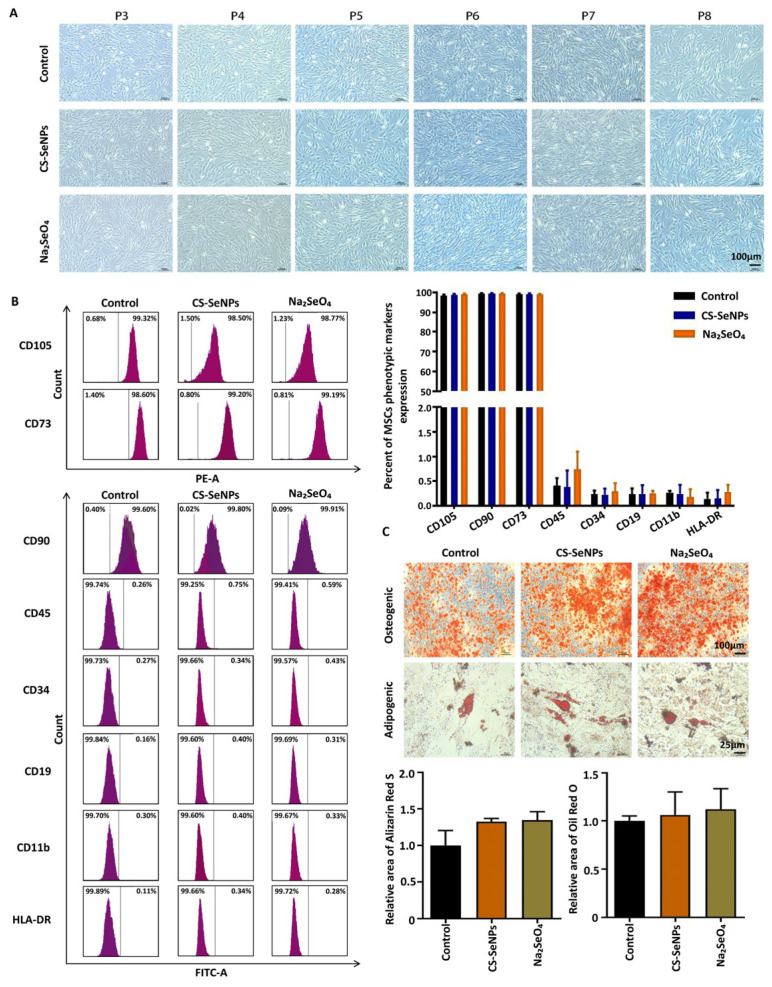
Analysis of the hUC-MSC phenotype and differentiation potential. (**A**) Morphological images of cells from the control and selenium compound groups were assessed. (**B**) Flow cytometry was conducted to analyze the phenotypic marker profiles of the hUC-MSCs. The data are from three independent biological replicates (n = 3) and are presented as the means ± SDs. (**C**) Oil red O staining and Alizarin red staining were used to detect the osteogenic and adipogenic differentiation of hUC-MSCs. The data are from three independent biological replicates (n = 3) and are presented as the means ± SDs.

To investigate the potential impact of these selenium agents on the basic characteristics of hUC-MSCs, we first examined the cellular morphology of hUC-MSCs after treatment with CS-SeNPs or Na_2_SeO_4_. The morphology of cells from the control and selenium compound groups at passages P3 to P8 is shown in Figure 2A. The size of the hUC-MSCs at passages P3 and P8 was relatively uniform. Similar to the cells from the control group, no obvious morphological differences were detected in the hUC-MSCs from the CS-SeNPs group and the Na_2_SeO_4_ group. Furthermore, the phenotypic marker profiles of the hUC-MSCs were analyzed at P3 via flow cytometry. The hUC-MSCs in the CS-SeNPs, Na_2_SeO_4_ and control groups presented expression of the phenotypic markers CD105 (98.48 ± 0.44 versus 98.86 ± 0.42 versus 99.19 ± 0.34), CD73 (99.05 ± 0.47 versus 99.02 ± 0.58 versus 99.20 ± 0.14) and CD90 (99.43 ± 0.26 versus 99.44 ± 0.49 versus 99.59 ± 0.31), and low expression of the negative markers CD45, CD34, CD19, CD11b and HLA-DR (Figure 2B). These data suggested that the expression of phenotypic markers was not affected by the selenium compounds.

We next examined the role of selenium compounds in hUC-MSC osteogenic and adipogenic differentiation via Alizarin Red staining and Oil Red O staining assays. Oil Red O staining revealed that the hUC-MSCs formed many neutral lipid droplets in the cytoplasm. Alizarin Red staining was used to assess mineral accumulation and bone nodule formation. The results revealed that hUC-MSCs cultured with or without selenium agents both possessed the potential to undergo adipogenic and osteogenic differentiation (Figure 2C).

### 3.3. Effects of Selenium Compounds on the Proliferation Capacity of hUC-MSCs

**Figure 3:**
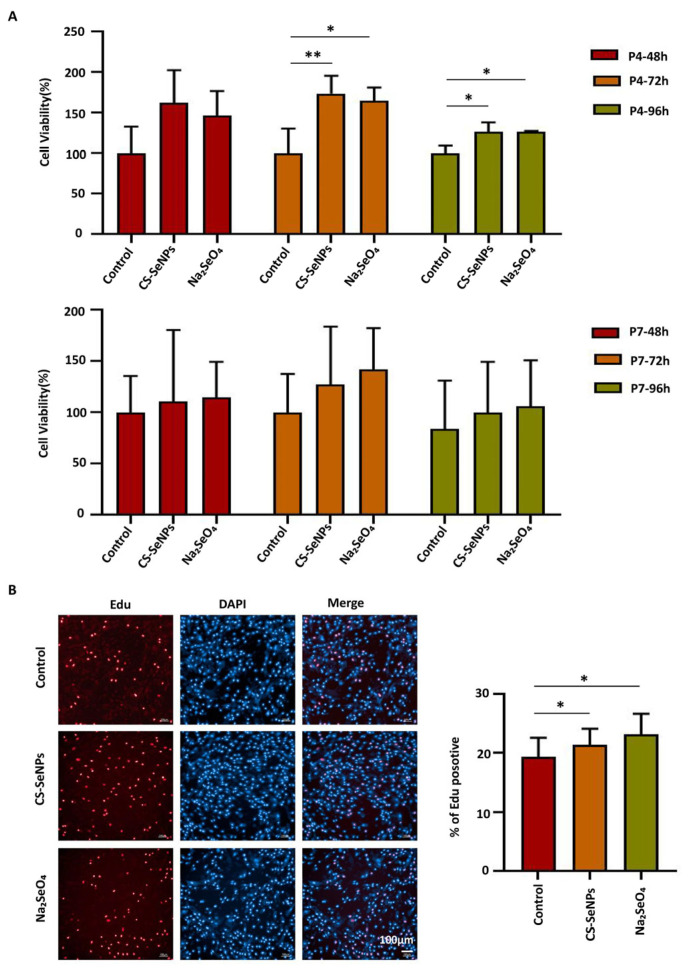
Effects of selenium compounds on the proliferative capacity of hUC-MSCs. (**A**) CCK8 assays were performed to assess the viability of the hUC-MSCs in the CS-SeNPs and Na_2_SeO_4_ groups. The data are from three independent biological replicates (n = 3) and are presented as the means ± SDs. (**B**) EdU assays were used to analyze the proliferation rate of hUC-MSCs in the CS-SeNPs and Na_2_SeO_4_ groups. The data are from three independent biological replicates (n = 3) and are presented as the means ± SDs. * *p* < 0.05, ** *p* < 0.01.

According to the previous results, we assessed the impact of culture on proliferation via the use of hUC-MSC medium or hUC-MSC medium supplemented with selenium compounds. As shown in Figure 3A, the viability of hUC-MSCs was significantly greater in the CS-SeNPs and Na_2_SeO_4_ groups than in the control group at both 72 h and 96 h in early passage P4, as determined by the CCK-8 assay. At 72 h, the viability reached 173.71 ± 22.12% and 164.70 ± 16.53% for the CS-SeNPs and Na_2_SeO_4_ groups, respectively, compared with that of the control (100.00 ± 30.60%). This promoting effect persisted at 96 h, with viability values of 126.52 ± 11.68% (CS-SeNPs) and 127.04 ± 0.66% (Na_2_SeO_4_) versus the control (100.00 ± 9.50%). In late passage P7, the viability of the hUC-MSCs in the selenium compound groups increased slightly compared with that in the control group, although the differences were not statistically significant (Figure 3A). Consistently, the results of the EdU assays revealed that the proliferation rate of P4 hUC-MSCs was significantly greater in the CS-SeNPs and Na_2_SeO_4_ groups than in the control group (19.38 ± 3.22%, 21.42 ± 2.71%, and 23.18 ± 3.47% for the control, CS-SeNPs, and Na_2_SeO_4_ groups, respectively) (Figure 3B). These results suggested that both CS-SeNPs and Na_2_SeO_4_ can effectively promote hUC-MSC proliferation at early passages.

### 3.4. Effects of CS-SeNPs and Na_2_SeO_4_ on the Cell Senescence and Oxidative Stress of hUC-MSCs

**Figure 4:**
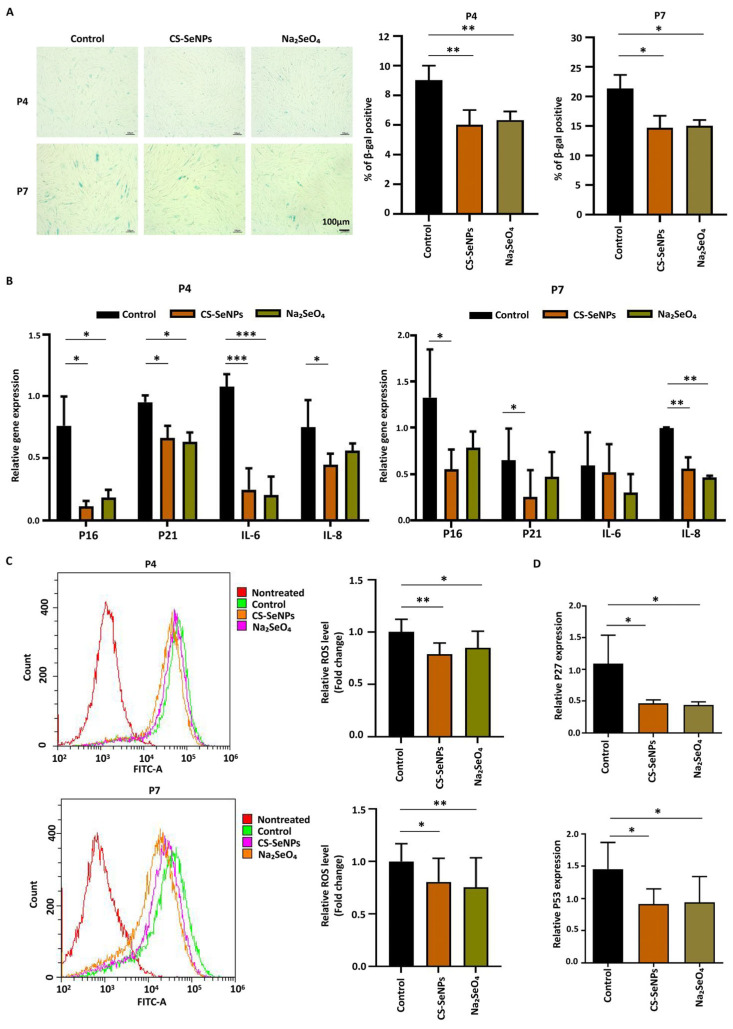
Effects of CS-SeNPs and Na_2_SeO_4_ on the oxidative stress and cell senescence of hUC-MSCs. (**A**) β-Galactosidase staining experiments were performed to compare the degree of senescence between the selenium compound groups and the control group. The data are from three independent biological replicates (n = 3) and are presented as the means ± SDs. (**B**) QPCR was performed to analyze the expression levels of key senescence markers, including *p16*, *p21*, *IL-6* and *IL-8*. The data are from three independent biological replicates (n = 3) and are presented as the means ± SDs. (**C**) The levels of ROS in hUC-MSCs cultured with CS-SeNPs, Na_2_SeO_4_, or the control group were measured using flow cytometry. The data are from five independent biological replicates (n = 5) and are presented as the means ± SDs. (**D**) QPCR assays were conducted to detect the expression levels of *p27* and *p53*. The data are from five independent biological replicates (n = 5) and are presented as the means ± SDs. * *p* < 0.05, ** *p* < 0.01, *** *p* < 0.001.

The long-term culture of hUC-MSCs leads to cellular senescence, a process characterized by a decrease in proliferative capacity, an increase in β-galactosidase activity, and the accumulation of ROS. Previous work suggested that selenium could not only improve cell survival but also reduce oxidative stress, preventing the senescence of hUC-MSCs [29,30]. Thus, we conducted β-galactosidase staining experiments to compare the degree of senescence between the selenium compound groups and the control group at passages P4 and P7. The presence of both CS-SeNPs and Na_2_SeO_4_ significantly reduced the percentage of β-gal-positive hUC-MSCs at both P4 (control: 9.00 ± 1.00% versus CS-SeNPs: 6.00 ± 1.00% versus Na_2_SeO_4_: 6.33 ± 0.58%) and P7 (control: 21.33 ± 2.31% versus CS-SeNPs: 14.67 ± 2.08% versus Na_2_SeO_4_: 15.00 ± 1.00%) (Figure 4A). Furthermore, we measured the expression of additional senescence markers in hUC-MSCs at passages P4 and P7 via qPCR. The results demonstrated a consistent downregulation trend across key markers of cell cycle arrest (*p16* and *p21*) and the senescence-associated secretory phenotype (*IL-6* and *IL-8*) upon selenium treatment (Figure 4B). Overall, these data indicated that selenium can reduce the senescence of hUC-MSCs.

Next, intracellular ROS levels were measured to assess the impact of selenium on hUC-MSCs. At both P4 and P7, the relative ROS level was significantly reduced in the CS-SeNPs and Na_2_SeO_4_ groups compared to the control group. Specifically, the values were 0.79 ± 0.11 (CS-SeNPs) and 0.85 ± 0.16 (Na_2_SeO_4_) at P4, and 0.80 ± 0.23 (CS-SeNPs) and 0.75 ± 0.28 (Na_2_SeO_4_) at P7, versus the control (1.00) (Figure 4C), confirming the potent antioxidant effects of these selenium forms. These findings suggested that the proliferation-promoting effect and anti-senescence effect of selenium on hUC-MSCs are correlated with their antioxidant properties.

To gain a deeper insight into the mechanisms driving the phenotypes of cellular proliferation and senescence, we examined the expression levels of *p27* and *p53* to explore the molecular changes associated with cellular proliferation and senescence. *P27* is an important cell cycle regulatory protein that can cause cell cycle arrest and inhibit cell proliferation. *P53* is a crucial regulatory gene in mediating cellular aging and the response to DNA replication damage. As shown in Figure 4D, the relative expression levels of *p27* and *p53* mRNAs were decreased in the cells cultured with the CS-SeNPs and Na_2_SeO_4_. Specifically, the expression of *p27* was significantly lower in the CS-SeNPs group (0.46 ± 0.05) and the Na_2_SeO_4_ group (0.44 ± 0.05) than in the control group (1.09 ± 0.45). A consistent decreasing trend was also observed for *p53* expression, which was lower in both the CS-SeNPs group (0.91 ± 0.23) and the Na_2_SeO_4_ group (0.94 ± 0.40) than in the control group (1.46 ± 0.41). These findings indicate that selenium can promote the proliferation and reduce the cellular senescence of hUC-MSCs by decreasing the levels of *p27*, *p53* and ROS.

## 4. Discussion

MSCs have been confirmed to have potential clinical application value in a variety of diseases because of their immune regulation, anti-inflammatory, and multipotent differentiation capabilities. To facilitate the rapid translation and implementation of this novel therapy, countries around the world have prioritized and supported the clinical application of MSCs, considering them as a key area for development. There are currently more than 1200 registered clinical trials involving MSCs and over 75,000 publications on MSCs [31,32]. Media formulated specifically for the cultivation of MSCs are essential for advancing the clinical application of MSCs.

Selenium, known for its antioxidant activity effect, has been extensively utilized in various studies. It was selected as a supplement to assess the effects of MSCs [33,34,35]. An increasing number of studies have highlighted the significant impact that these selenium compounds may have on enhancing the efficacy of MSCs, indicating their potential as valuable adjuncts in the process of MSC cultivation. Different selenium compounds are metabolized via distinct pathways within cells, consequently exerting unique effects and mechanisms on cell growth [36,37]. It is essential to conduct a comprehensive investigation into the safety and pharmacodynamic effects of different doses and compounds of selenium to optimize its use in cell culture. The present study demonstrated that the application of selenium to hUC-MSCs resulted in increased cell proliferation, which aligns with previous studies [18,19]. However, different forms of selenium compounds, including organic selenium, inorganic selenium and SeNPs, have different effects on the proliferation capacity of hUC-MSCs. All these selenium compounds can promote the proliferation of hUC-MSCs at specific concentrations. Among the diverse selenium-based compounds, we found that, compared with organic selenium (Ebselen and SeMet), inorganic selenium (Na_2_SeO_4_) and SeNPs (CS-SeNPs) exhibit greater bioavailability in the process of hUC-MSC cultivation, but organic selenium (Ebselen and SeMet) has lower toxicity. The observed differences are likely attributable to variations in uptake efficiency and metabolic pathways associated with the distinct chemical forms of different selenium compounds [38]. CS-SeNPs enable enhanced cellular uptake and controlled selenium release, thereby promoting sustained proliferation [39]. Na_2_SeO_4_ is rapidly metabolized into selenoproteins, whereas SeMet is nonspecifically incorporated into proteins, limiting its availability for functional selenoprotein synthesis. Ebselen, which lacks direct selenium donation, has relatively weak proliferative effects. Studies indicate that sodium selenite supports stress erythropoiesis with minimal inflammation, whereas high-dose SeMet increases inflammatory responses [40]. Chen et al. [41] reported that SeMet was more effective than ebselen in restoring the viability of ethanol/acetaldehyde-injured MSCs. The specific mechanisms involved require further analysis of the intracellular selenium content and speciation.

The long-term culture of MSCs leads to cellular senescence, which compromises both their biological properties and therapeutic functions. For example, Wang et al. demonstrated that long-term cultured MSCs exhibit decreased proliferation and differentiation, reduced immunosuppression abilities, and widespread alterations in gene expression patterns after long-term culture [42]. Cell senescence is influenced by multiple factors, including epigenetic modifications, DNA damage, and the accumulation of ROS. ROS play dual roles in MSC culture. An appropriate amount of ROS can function as intracellular signaling molecules, promoting cell proliferation, preventing cell senescence and regulating various cellular signaling pathways [43,44,45]. Conversely, excessive ROS cause oxidative stress and damage cellular cell membranes, proteins, and DNA, potentially inhibiting cell proliferation and even leading to cell death. Studies have shown that selenium supplementation can extend the lifespan of MSCs and enhance their paracrine effects on wound healing and tissue regeneration by inhibiting ROS-mediated aging [18]. Therefore, understanding and controlling ROS levels is a key factor in enhancing the therapeutic efficacy and safety of MSCs in laboratory culture and clinical applications. Our findings revealed that CS-SeNPs and Na_2_SeO_4_-treated hUC-MSCs can reduce the senescence of MSCs via β-galactosidase staining experiments. Both selenium forms significantly reduced intracellular ROS levels and enhanced the antioxidant activity of the cells. Our results demonstrate that appropriate selenium supplementation enhances the cellular antioxidant capacity, helping to maintain ROS at healthy levels, and thus promoting cell function and survival. These findings are consistent with a growing body of evidence highlighting the role of selenium in oxidative stress-related pathologies. For example, one study showed that SeNPs rebalance redox homeostasis by activating GPx1, thereby reducing ROS levels and ameliorating lumbar disc degeneration [45]. In addition, Li et al. [29] reported that transformed SeNPs activate GPx1 to reduce mitochondrial damage and ROS overproduction, reversing HAdV-14-induced oxidative damage.

To elucidate the mechanisms by which selenium treatment affects the proliferative capacity of MSCs, we analyzed the expression levels of the proliferation-associated gene *p27* and the senescence-associated gene *p53* via qPCR analysis. We observed that, in response to selenium treatment, *p27* and *p53* were dramatically downregulated. *P27* acts as a negative regulator of the cell cycle by inhibiting cyclin-CDK complexes, thereby blocking the phosphorylation of key substrates required for DNA replication and cell division. Its downregulation is frequently associated with the activation of the PI3K/Akt survival pathway. This signaling cascade leads to the suppression of *p27* transcription, promoting proliferation [46]. *P53* is a crucial regulatory gene that mediates cellular aging and the response to DNA replication damage. Research has shown that, although the molecular mechanisms that activate aging signaling pathways vary, regardless of whether stem cell aging is induced by intrinsic or extrinsic factors, it ultimately exerts its effects primarily through the regulation of cell cycle-related proteins such as *p53*. When *p53* expression is abnormal, various characteristics associated with cellular aging can be triggered. The decrease in *p53*, potentially achieved through a selenium-driven antioxidant response (e.g., the Nrf2 pathway) that reduces ROS and disrupts a *p53*-ROS feedback loop, alleviates the cellular senescence program [47,48]. Together with *p27* downregulation, these changes collectively establish a molecular environment favorable for proliferation.

This study has several limitations. First, although we observed characteristics of cellular senescence, the senescence markers were analyzed exclusively at the mRNA level by qPCR, and we did not validate the DNA damage marker γH2AX. Second, our analysis was confined to early passages (P4 and P7). While this allowed us to capture the initial onset of senescence, it may not fully represent the more robust and mature senescent phenotype typically observed in later passages. Future work will extend the culture timeline to characterize the progression of the senescent state more comprehensively.

In conclusion, our study indicated that selenium can promote the proliferation of hUC-MSCs at specific concentrations. However, different forms of selenium compounds, including organic selenium, inorganic selenium and SeNPs, have different effects on the proliferative capacity of hUC-MSCs. Na_2_SeO_4_ and CS-SeNPs exhibit greater bioavailability in the process of hUC-MSC cultivation, but ebselen and SeMet have lower toxicity. Our results indicate that Na_2_SeO_4_ and CS-SeNPs enhance hUC-MSC proliferation and reduce senescence by inhibiting the levels of *p27*, *p53* and ROS, suggesting potential applications in the manufacturing of clinical-grade hUC-MSCs. However, further in vivo and mechanistic studies are warranted to validate its safety and efficacy.

## Figures and Tables

**Table 1:** QPCR primers sequences used in this study.

Gene	Forward Primers (5′-3′)	Reverse Primers (5′-3′)
*P27*	AACGTGCGAGTGTCTAACGG	CCCTCTAGGGGTTTGTGATTCT
*P53*	GAGGTTGGCTCTGACTGTACC	TCCGTCCCAGTAGATTACCAC
*P16*	CGTACCCCGATTCAGGTG	ACCAGCGTGTCCAGGAAG
*P21*	GGCAGACCAGCCTGACAGAT	TTCAGGGTTTTCTCTTGCAGAAG
*IL-6*	AGACAAAGCCAGAGTCCTTC	TTCTGTGACTCCAGCTTATC
*IL-8*	GAGAGTGATTGAGAGTGGACCAC	CACAACCCTCTGCACCCAGTTT
*β* *-ACTIN*	TGAAGATCAAGATCATTGCTCCTC	AACTAAGTCATAGTCCGCCTAGAAG

## Data Availability

All data relevant to the study are included in the article.
